# Applying Time-Dependent Attributes to Represent Demand in Road Mass Transit Systems

**DOI:** 10.3390/e20020133

**Published:** 2018-02-20

**Authors:** Teresa Cristóbal, Gabino Padrón, Javier Lorenzo-Navarro, Alexis Quesada-Arencibia, Carmelo R. García

**Affiliations:** 1 Institute for Cybernetics, Campus de Tafira, Las Palmas de Gran Canaria, University of Las Palmas de Gran Canaria, 35017 Las Palmas, Spain; 2 University Institute of Intelligent Systems and Numeric Applications in Engineering, Campus de Tafira, Las Palmas de Gran Canaria, University of Las Palmas de Gran Canaria, 35017 Las Palmas, Spain

**Keywords:** clustering, entropy, attribute creation, data mining, intelligent transport systems, mass transit systems, demand

## Abstract

The development of efficient mass transit systems that provide quality of service is a major challenge for modern societies. To meet this challenge, it is essential to understand user demand. This article proposes using new time-dependent attributes to represent demand, attributes that differ from those that have traditionally been used in the design and planning of this type of transit system. Data mining was used to obtain these new attributes; they were created using clustering techniques, and their quality evaluated with the Shannon entropy function and with neural networks. The methodology was implemented on an intercity public transport company and the results demonstrate that the attributes obtained offer a more precise understanding of demand and enable predictions to be made with acceptable precision.

## 1. Introduction

According to the International Energy Agency, there were an estimated 870 million passenger light-duty vehicles on our roads worldwide in 2011, a figure that is projected to grow to 1.7 million in 2035 [1]. This type of mobility, based on private vehicles, is resulting in the deterioration of health, the environment and safety on the roads. To illustrate this problem, the World Health Organisation estimates that approximately 3 million people die every year due to health problems caused by pollution [2] and in the European Union 250,000 people are victims of traffic accidents, and about 10% of these accidents are fatal [3]. Large-scale public road transport systems are an effective means to respond to mobility needs in a way that is safer and more respectful towards our health and the environment. For this reason, the development of efficient transportation systems that provide quality of service is a priority for the authorities and for transport agencies. Consequently, models and techniques that contribute to the development of efficient systems and that provide quality of service are a topic of great interest for the academic community.

In the context of large-scale road transport systems, knowledge of demand is important, as it must be taken into account when rolling out transport systems that provide quality of service to users and that are efficient from the point of view of resource requirements. Because demand in this type of system is variable, planning will vary depending on time-dependent characteristics, such as: time of year, month and day, etc. This article proposes a new type of time-dependent attribute that will enable a more precise understanding of demand, and describes how to obtain the attributes systematically by means of data mining. 

This paper makes three main contributions. The first is the proposal of a new class of attributes to represent the behaviour of demand. These attributes are sensitive to the time periods that affect demand (time of year, month, day of the week, work day, public holiday, time of the day, etc.). They provide information about demand and therefore help to develop schedule plans adapted to the behaviour and to predict future demand. The second contribution is the fact that the proposed attributes are the result of a clustering process, in which each cluster represents a different demand pattern occurring in different instants of time. This is a novel approach to classification. The third contribution is the fact that it was developed in a public road transport system planned by timetable, an approach used in intercity or long-distance transport. Most of the studies that have addressed problems related to the subject matter of this paper have been developed in the context of systems planned by frequency of stops, which is the case of urban transport systems.

The rest of this article is organised into five sections. The following section will review studies related to the proposed methodology, which will serve to contextualise and highlight its interest and originality. The methodology, based on data mining, used to obtain the new representation model is described in the third section. The fourth section is devoted to presenting the results obtained in a real use case, as the representation model has already been implemented in a public passenger transport company. The results are discussed in the fifth section. Finally, the conclusions are presented in the sixth section.

## 2. Related Studies 

The review in this section aims to meet two objectives. The first is to highlight the importance of demand and models that represent it when developing mass transit systems that provide a quality service. The second is to review the use of data mining in public transport, setting out the goals that were pursued and the techniques used.

The efficiency and quality of service in mass transit systems is an important challenge for the authorities and for transport agencies. This challenge should be addressed through the use of methodologies and techniques that allow optimal design of the transport network, of planning and operations monitoring. Moreira [4] conducted an exhaustive review of these techniques and methodologies, often in relation to demand. 

The solution to the problem of designing an optimal transport network resides in striking a balance between normally opposed factors. A first, essential factor is responding to the mobility needs of users. Another factor, related to the viability and sustainability of the network, is the resources that are used. A final factor is the time spent travelling: travel time. In the various methodologies proposed for an optimal design of the transport network, demand is a factor to consider, and the most widely-used form of representation is the origin-destination matrix (O–D matrix). This representation method consists of a matrix in which each row represents the trip origin nodes and the columns the destination nodes. Thus, the value *M*(*x*,*y*) represents the number of travellers who have travelled from node *x* to node *y* in a given period of time (Desaulniers [5]). In this context, different demand models have been developed, which can be classified into two categories: deterministic and stochastic. Deterministic models have been used to predict demand over a period of time [6]. Stochastic models are a variant of deterministic models. They are used to predict the demand variations that occur daily in the transport network, for example, and are represented by the O–D matrix containing the mean value and the standard deviation [7]. An alternative approach to determining passenger flow using the O–D matrix was proposed by Berbey [8], using fuzzy logic to determine the values of its elements on a public transport metro system.

In the context of mass transit systems by road, the purpose of an operations planning design problem is to specify a sequence of operations to be carried out by vehicles in order to create a quality transport service. In this context, quality of service means adherence to the planned schedules and reduction of traveller waiting times at stops. There are two types of operations planning: planning by frequency of stops and planning by timetable. Planning by frequency of stops is used in urban transport and the models used generally do not take into account demand quantification data; they assume that the traveller goes to stops randomly. An exception to this approach is the work of Patnaik [9], who used data mining, specifically clustering techniques and decision trees, to develop optimal operations plans, using the cumulative number of passengers travelling in a vehicle on a trip and the passing times at each stop on the route. Using machine learning techniques—specifically, clustering techniques and decision rules—Mendes-Moreira [10] described a framework that uses automatic vehicle location systems (AVL) data to test whether the established schedule plan fits the conditions in the design of the transport network planned by frequency. Khiari [11] also used clustering techniques based on Gaussian Mixture Models (GMM) to set bus schedule coverage in a context of planning by frequency, using AVL and APC data. Although these two studies made use of clustering techniques, the techniques that they employed were different, as were the data used (location and passenger counting). When the route is planned by timetable, it is assumed that the arrival of passengers at the stop is a function of the scheduled passing time, namely, demand conditioned by service (Furth [12]); it is therefore necessary to predict the number of passengers that will be at the stops on a route at any given time.

On public road transport systems, operations monitoring aims to provide the appropriate response, usually in real time, to incidents that occur on the transport network and this may affect adherence to scheduled operations and quality of service. According to Dessouky [13], the type of information required by the methods implemented by these strategies are: planning of vehicle operations, information on vehicle headways, a prediction of headway times and a prediction of the passengers waiting at each of the stops on the route. For this type of method, it is assumed that, in general, the behaviour of the transport network is stochastic, using probabilistic models to represent different relevant factors involved in operations control. Hadas [14] proposed an optimisation method with the goal of improving the reliability of public transport services by optimally reducing the transfer times required in transport network operations, using O–D matrices obtained at a given time as input data in simulations to verify the validity of the proposal. Sáez [15] proposed a predictive control method, the objective of which was to minimise the passenger’s travel time, modelling the passengers that boarded and alighted from the buses with O–D matrices obtained from historical data in different time periods.

Technological advances in public road transport systems, especially in on-board systems, sensor networks and personal devices, have allowed for a large amount of data. These data—from payment systems, automatic passenger counting systems (APC) and automatic vehicle location systems (AVL)—have allowed researchers to use data mining techniques and, especially, classification techniques to acquire information on the behaviour of the traveller and the transport network. In the case of the data from payment systems, particularly those provided by systems based on contactless cards, Agar [16] defined market segments within public transport users. Lathia [17] proposed a system to predict individual travel times and Du [18] also used the data from automatic payment systems, together with socio-demographic data, to obtain behaviour mobility patterns that would enable the demand for public transport services in areas of urban expansion to be estimated.

Using AVL systems as a data source, Sun [19] proposed a system to predict the arrival time of buses at stops by combining clustering techniques with other methods. Based on positioning data and, more specifically, on data generated by GPS devices on taxis, some studies have generated mobility patterns in urban areas. Yuan [20], starting from segmentation of such a space based on its main routes, and considering the points of interest located in each of those segments (restaurants, shopping centres, residential buildings, etc.) and the mobility patterns obtained for all the trips, obtained a set of functions for each of these segments that characterise this mobility. To this end, a model inspired by probabilistic topic models [21] was used to recognise content in documents. Zhao [22] segmented the urban space by means of the quad-tree division, considering only the population mobility data generated in taxis, without prior information on infrastructures, since a strong correlation had been found between infrastructure and the trips made by this means of transport.

## 3. Methodology

This paper was developed in the context of public intercity or long-distance road transport systems, which, as previously stated, are scheduled by timetable. In this type of system, operations planning is carried out using attributes that represent periods of time that must be adapted to variations in demand in order to optimise the resources to be used. Examples of commonly used time periods are: working days, public holidays, weekends, school day, etc. Therefore, information about the different periods that affect demand on the routes of the transport network is a basic requirement for optimal planning. For example, demand on one route will also vary on the same day, depending on the time of day. For this reason, scheduling should also use attributes that represent period types such as, for example, peak and off-peak. Therefore, to design an optimal schedule and to monitor that schedule, it is necessary to know how demand varies on a given day, and this demand depends on the time of day. 

This paper proposes the use of a new type of attribute to classify demand. For a given route, each attribute value represents a demand pattern that may occur in different periods of time, such as: time of year, month, week, day of the week, time of day, etc. The aim is to obtain more precise knowledge of how demand varies over time, so that this knowledge can be used to optimise the planning and monitoring of operations. This section presents the methodology used to obtain these attributes. Based on data mining, it uses clustering techniques to generate them, entropy functions to evaluate the amount of information that they provide and neural networks to analyse their capacity to predict demand.

**Figure 1:**
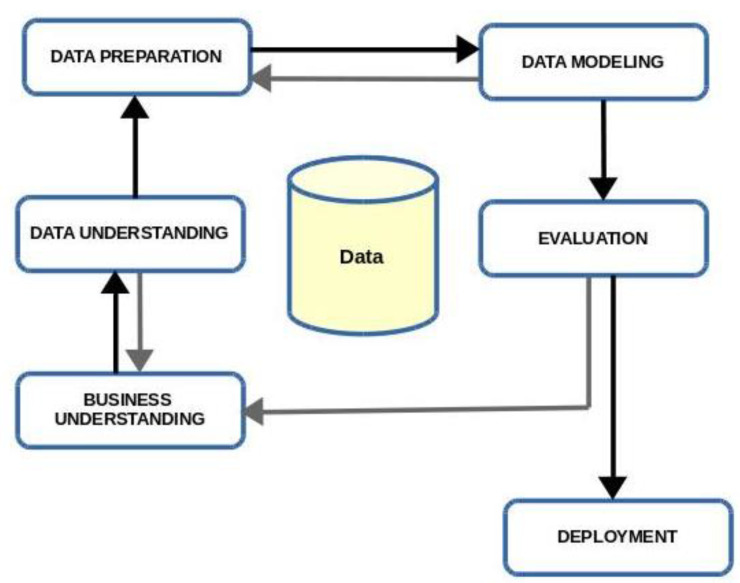
Process cycle of the CRoss-Industry Standard Process for Data Mining (CRISP-DM) model.

The methodology used was the process-oriented methodology called CRoss-Industry Standard Process for Data Mining (CRISP-DM) [23]. With this methodology, the processes are clustered in different phases to form the main cycle (see Figure 1). As can be seen, the process is not strictly sequential: the phases are interrelated and may move forward (black lines in the diagram) or backward (grey lines) depending on whether or not the objectives are attained. The purpose of the initial phase, Business Understanding, is to determine the scope of the problem and set the main data mining project targets. The data understanding phase is designed to analyse the available data and solve the problems that may be detected. The data preparation phase comprises all the tasks related to the construction of the final dataset that will be subjected to the procedures of the next phase, Modelling, in which different methods and parameters are tested, the validity of the results is evaluated and, if necessary, the data preparation phase begins anew. The evaluation phase verifies that the results respond to the needs of the organisation, which were defined in the first phase of the project, reconsidering them if necessary. Depending on the project requirements, tasks related to the final phase, Deployment, may differ, depending on the nature of the project, and involves incorporating the acquired information in the form of a report or as a new procedure in the organisation.

This study mainly encompasses two of these phases: Data preparation and Modelling. In the first, Data Preparation, the data that form the basis for this study (listed below) were merged and complemented, and the dataset for training and testing the neural networks was defined. In the second, Modelling, the modelling tools were incorporated, and different methods and parameters were tested to achieve the desired results. 

The implementation of this methodology is described below. The public transport company Global Salcai-Utinsa collaborated with this study by providing access to their data. It is a company that operates on the island of Gran Canaria (Canary Islands, Spain) and is the main intercity transport company on this island; it has a fleet of 304 vehicles operating on a transport network with 2686 stops, 110 different routes and 2395 daily routes. Annually, its vehicles travel 28,897,002 kilometres and transport 19,284,378 passengers [24].

### 3.1. Data Preparation Phase

The input data used for this methodology are intrinsic to transport services and do not require external sources. Only records that are considered in the operational systems (operations and payments made on vehicles) are used, and others, such as vehicle location, are not included. Therefore, the method may be reproduced on most public transport companies since it does not require very sophisticated technological means. The data used came from the systems installed on the vehicles of the fleet. These systems record all the relevant events that occur on the vehicles. For this methodology, the data records are used to record the beginning and end of each line service, the change of stop, and the payment made by a traveller on the vehicle, which can be in cash or by means of a contactless card. Based on the records associated with these events, the objective is to obtain new attributes that describe the different passenger demand patterns and thus be able to estimate them. Considering the different periods of time that are used in the planning and monitoring of operations, to classify the demand patterns, it was considered sufficient to define three different time scales:

- Week of the year, to detect variations in demand depending on the time of year.
- Day of the week, to detect variations in demand depending on the day of the week.
- Time of day, to detect variations in demand depending on the time of day.

The events and data used in this study were as follows:

- Line service start event: the service start date and time, the vehicle that performed the line service, the route and the trip number.
- Line service end event: the line service end date and time, the vehicle that performed the line service, the route and the trip number.
- Stop change event: the date and time of the stop change, the vehicle that recorded the change of stop, the stop, route and trip number.
- Cash payment for travel event: the date and time of payment, the vehicle, the type of fare applied, the number of passengers, the origin stop of the trip, the destination stop of the trip, the cost, the route and the trip number.
- Contactless card payment for travel event: the date and time of payment, the vehicle, the type of fare applied, the number of passengers, the origin stop of the trip, the destination stop of the trip, the cost, the route and the trip number.

It should be noted that, although the events and data directly related to the purpose of this study are those that describe each trip (origin, destination, date, time and number of travellers), all other events and data have been used for validation purposes. It should also be noted that these data are operational data that are usually recorded by public transport companies; therefore, the proposed methodology may be applied to most intercity or long-distance transport companies without the need for any specific technological installation.

From these data, the initial dataset of records was constructed, specifying the point of origin—stop *P_o_* on the transport network—the destination—stop *P_d_* on the network—the demand and the period of time, *T*, to be analysed. With these initial specifications and accessing the transport operator’s database, all the records that represent the line services from the period *T* and that passed through points *P_o_* and *P_d_* were obtained. 

Once the integrity of the initial dataset was guaranteed, it was processed and two datasets were obtained. The first one was used to obtain the new attributes. The second was used as the training and test dataset of the demand prediction neural network. In Table 1, the record structure of the first dataset is represented; the meaning of the fields is as follows:

- *P_o_* origin stop of the demand to be analysed.
- *P_d_* destination stop of the demand to be analysed.
- *W* number of the week of the year, assuming that one year has 52 or 53 weeks.
- *D* day of the week: 1 Monday, 2 Tuesday, ..., 7 Sunday.
- *A_W,D,H_* total demand on day *D*, of week *W*, at time *H* from stop *P_o_* to stop *P_d_*. *H_0_* indicates the first hour of the day analysed and *H_f_* indicates the last hour of the day analysed.

The steps followed to obtain the dataset for classifying demand are described below:

1. Data were obtained from the transport database relating to the bus lines operating on the routes that include the trip to be analysed in the time period *T*. This dataset was called *L*.
2. For each bus line in dataset *L*, data on the line services that operated during the time period *T* were obtained. This dataset was called *SL*.
3. For each line service in *SL*, data for all the payments made by the travellers for whom the origin stop was *P_o_* and the destination stop *P_d_* were obtained. This dataset was called *MT*.

For each payment record from the *MT* dataset, using the recorded date and time of payment, the movement was added to the corresponding records of the dataset for classifying demand.

The record structure of the training and test datasets for evaluation of the new attribute, based on its use in the prediction of demand using a neural network, is presented in Table 2. The meaning of the fields is as follows:

- *P_o_* origin stop of the demand analysed.
- *P_d_* destination stop of the demand analysed.
- *W* number of the week of the year, assuming that one year has 52 or 53 weeks.
- *D* day of the week: 1 Monday, 2 Tuesday, ..., 7 Sunday.
- *F_Po_* indicates public holiday at the origin stop.
- *F_Pd_* indicates public holiday at the destination stop.
- *C* is the new attribute, the value of which corresponds to a demand pattern for the values *P_o_*, *P_d_*, *W* and *D*, obtained in the clustering process applied to the dataset for classifying demand. The demand pattern is the identifier of the cluster.
- *N* is the number of passengers to be forecast, who go from stop *P_o_* to stop *P_d_*.

### 3.2. Modelling Phase

This phase consisted of two processes. The objective of the first was to obtain the new attributes that describe demand and the purpose of the second was to evaluate the attributes that were generated.

The task of obtaining the attributes was carried out using clustering techniques in order to group the data according to their similarity, thus enabling the creation of different categories. Specifically, for each pair of stops, a subset of data was generated that exclusively contained the demand data represented in Table 1 (*A_W,D,H0_*, *A_W,D,H1_*, ..., *A_W,D,Hf_*). Each of the mentioned subsets was segmented into 2 to *k* clusters giving rise to new *k*-1 attributes, so that each value of the attribute corresponds to each of the clusters, which represent different demand patterns. There are many segmentation algorithms that Xu [25] classified depending on methodology and philosophy: those that use measures of distance and similarity, those based on quadratic error, on graph theory, hierarchical, and others. For the purposes of this study, segmentation techniques based on quadratic error were used since they are capable of handling large datasets that are frequently used in the context of transport, specifically the *K*-medoids algorithm [26], because it is one of the most robust against noise. A medoid may be defined as the object of a cluster whose average dissimilarity to all objects in the cluster is minimal. It is the point located the closest to the centre in the whole cluster.

The validity of a solution in a clustering problem is evaluated using validity indices. Following the classification proposed by Aldana-Bobadilla [27], these indices may be classified into three categories: external indices, which measure the extent to which cluster labels match externally-supplied class labels; internal indices, which measure the intrinsic information of each dataset, and relative indices, which are used to compare several different clustering solutions. For the purposes of this study, an internal index was chosen to measure the quality of the clusters in the first instance: the silhouette function [28]. This measures the consistency of the cluster based on a comparison of the tightness and separation of the elements of each segment generated and is computed by the following formula:

(1) $$s\left(i\right)=\{\begin{array}{c} 1-\frac{a\left(i\right)}{b\left(i\right)},if\;a\left(i\right)<b\left(i\right)\\ 0,if\;a\left(i\right)=b\left(i\right)\\ \frac{b\left(i\right)}{a\left(i\right)}-1,if\;b\left(i\right)<a\left(i\right) \end{array}$$

In Formula (1), *a*(*i*) is the average distance from object *i* to the other objects within the cluster and *b*(*i*) is the smallest average distance from *i* to all the objects of each of the clusters to which *i* does not belong.

Considering different authors, such as Lathia [29], who indicated that it is not always the case that the optimal value resulting from applying various methods to define the cluster number is the most appropriate, two additional evaluations were carried out to analyse the result of the segmentations. The first—independent criterion—consisted in evaluating the new attributes based on the intrinsic characteristics of the data. To this end, mutual information based on Shannon entropy was used, as expressed in Formula 2, which, in this case, is defined by the difference in uncertainty about the number of passengers not incorporated and incorporating the new attribute:

(2) I(Class;Attribute)=H(Class)+H(Attribute)−H(Class,Attribute)

**Figure 2:**
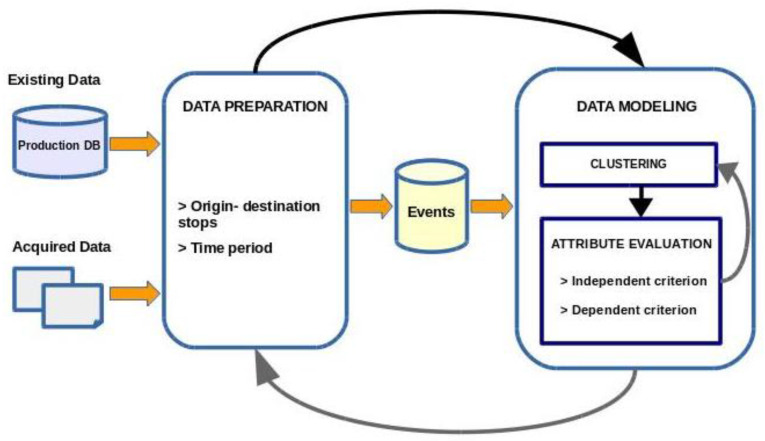
General scheme of processes in the evaluation of the new attributes.

The second evaluation method—dependent criterion—consisted in applying a data mining algorithm to ascertain the effect of the attributes and to select them. In our case, a neural network was trained using the indicated data to predict demand and the attribute was evaluated for the error generated with the test dataset. Neural networks for the estimation of demand are commonly used in the field of transport, due to their ability to process multidimensional data, their learning capacity and their predictive capacity [30]. Evidently, this second method is more costly in computational terms. The set of procedures involved is illustrated in Figure 2. This figure shows the main processes of this study, framed within the Data Preparation and Modelling phases:

- Data Preparation phase. Depending on the defined time granularity, creation of the datasets to be modelled from the origin-destination matrices.
- Data Modelling phase, divided in two main tasks: generation and evaluation of 2–*k* clusters, using an independent criterion and a dependent criterion.

## 4. Results

The methodology described was implemented in the intercity public transport company Global Salcai-Utinsa. It was used to study demand between three pairs of origin-destination stops on widely used routes by passengers of this company. Since demand was being analysed, six trips were studied for each pair of origin-destination stops, in both directions (outbound and inbound). The four stops were chosen according to different types of demand that should exist a priori depending on the socio-economic characteristics of the geographical areas in which they are located. These stops are described below:

- Stop identified with code 0. This stop corresponds to the main station in the city of Las Palmas de Gran Canaria, the capital of the island and the most populated municipality, which has the greatest number of public and private service centres.
- Stop identified with code 11. This stop corresponds to the main stop in the municipality of San Bartolomé de Tirajana. This is the biggest tourist municipality on the island of Gran Canaria; according to records, in 2016 it had 88,297 tourist beds [31].
- Stop identified with code 66. This stop corresponds to the main stop in the municipality of Santa Brígida. This municipality is a dormitory town for the capital of the island and its per-capita income is the highest on the island.
- Stop identified with code 99. This stop corresponds to Gran Canaria Airport. In 2016, this airport was used by 12,093,646 passengers, and is ranked fifth in the list of Spanish airports by the number of passengers [32].

From these stops, the following pairs of origin-destination stops were selected to analyse demand on the inbound and outbound trips:

- Trips between stops 0 and 66: trip from stop 0 to stop 66 (0–66) and trip from stop 66 to stop 0 (66–0).
- Trips between stops 0 and 11: trip from stop 0 to stop 11 (0–11) and trip from stop 11 to stop 0 (11–0).
- Trips between stops 99 and 11: trip from stop 99 to stop 11 (99–11) and trip from stop 11 to stop 99 (11–99).

With regard to the tools used, in the data preparation phase, Oracle was used for the database system and Pentaho for integration and visualisation. In the Modelling phase, the RStudio framework was used; more specifically, the Cluster [33], FSelector [34] and Neuralnet [35] modules. The data related to demand (*A_W,D,H_* in the dataset for classifying demand and *N* in the dataset for evaluating demand) were scaled in the processes of clustering and prediction with neural networks, using the maximum and minimum values for each of the routes. In the process of clustering, the metric used to calculate the dissimilarities between observations was the Euclidean distance, and the medoids were not initially specified. Nor were the weights of the neurons of the hidden layer initialised in the processes of creating the neural networks, where the differentiable error function used was the sum of squared errors.

### 4.1. Creation of the New Attribute

The results obtained in the creation phase of the new attributes are described below. As stated above, the structure of the data used is illustrated in Table 1. An origin–destination matrix was generated for each of the analysed routes, based on the direct or contactless card payment records of the passengers that made this trip, regardless of the bus line, between the first and final date of the analysis and during a specific time period. The period analysed was all of 2015, from 6:00 a.m. to 10:00 p.m. Table 3 shows the number of passengers on the trips analysed according to the means of payment used. From these initial specifications to define the period of analysis and considering the meaning of the data fields in Table 1 (dataset for classifying demand), the following values are used:

- *P_o_*–*P_d_* may have the following values: 0–66, 66–0, 0–11, 11–0, 99–11 and 11–99.
- *W* has a value of between 1 and 53, since 2015 had 53 weeks.
- *D* has a value of between 1 and 7.
- *A_W,D,H_* total number of passengers on day *D*, of week *W*, at time *H* from stop *P_o_* to stop *P_d_*. *H_0_* indicates the first hour of the day analysed (06:00) and *H_f_* indicates the last hour of the day analysed (22:00).

From the dataset for each trip, comprising the values indicated in Table 1 (*A_W,D,H0_*, *A_W,D,H1_*, ..., *A_W,D,Hf_*), seven different clusters were created according to the number of segments considered, from 2 to 8, using the *K*-medoids algorithm. The consistency of each of the seven clusters was evaluated with the silhouette function.

**Figure 3:**
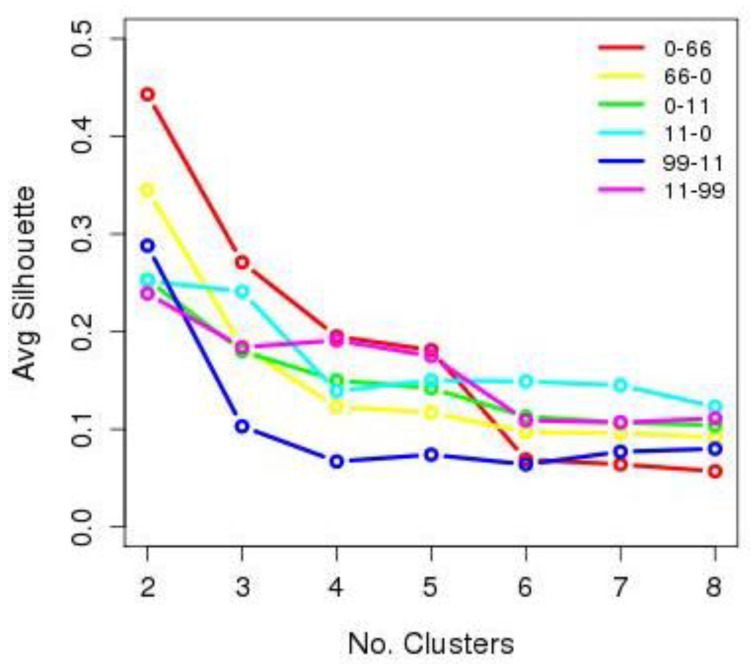
Result of the consistency analysis using the silhouette function. The horizontal axis represents the number of clusters considered in each clustering process. The average values obtained with the silhouette function are represented on the vertical axis.

Figure 3 shows the mean value for the silhouette function [26] obtained in each of the seven clusters for each of the analysed routes. The figure shows that the groupings of 2, 3 and 4 clusters are those that obtain higher consistency values. Owing to this result, only the new attributes resulting from these three cluster groups were evaluated; these new attributes were named *K2*, *K3* and *K4*.

**Figure 4:**
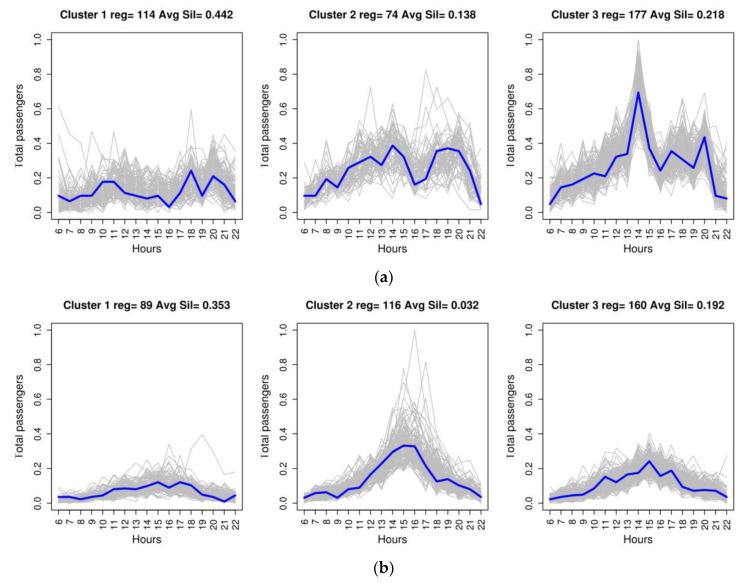

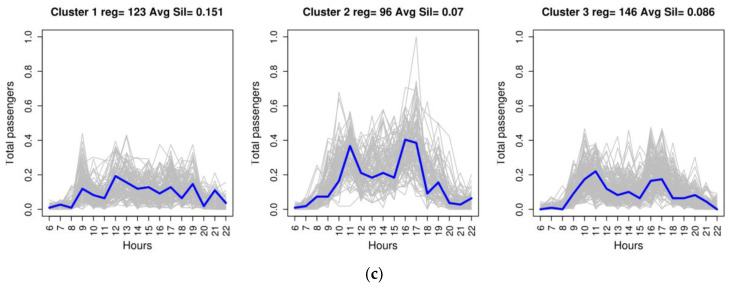
Clustering of three of the trips analysed. The horizontal axis represents the time of the day, from 6:00 a.m. to 10:00 p.m. The vertical axis represents the number of passengers, normalised. In the upper part of the graph for each cluster, the number of data records for that cluster and the consistency value of the cluster obtained with the silhouette function are indicated: (**a**) Clusters 1, 2 and 3 of Trip 0–66; (**b**) Clusters 1, 2 and 3 of Trip 0–11; and (**c**) Clusters 1, 2 and 3 of Trip 99–11.

Figure 4 illustrates the clustering result with three clusters for three of the six routes analysed, with the representative medoid of each cluster in blue.

This task gave a relationship between the fields *P_0_*, *P_d_*, *W* and *D* with the new attributes, such that, in the next evaluation phase, the value of said attribute was assigned to the *C* field of the corresponding data record of Table 2. 

### 4.2. Evaluation of the New Attributes.

The new attributes were evaluated according to two different criteria. The first applied criterion was the independent criterion, which consists of evaluating the information gain using the Shannon entropy function. To do this, the value of this gain was calculated for each of the attributes frequently used in planning (month, week of the year, day of the week and public holiday) and the new attributes (*K2*, *K3* and *K4*) with respect to the number of passengers class (attribute *N* of the data records from the learning and test datasets represented in Table 2). 

**Figure 5:**
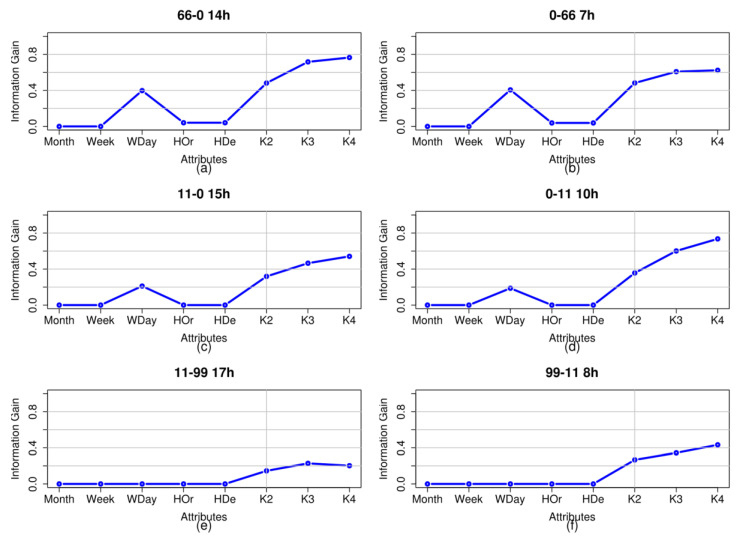
Shannon entropy values for the classic attributes and the new attributes against the number of passengers class for each of the trips analysed in the peak time period for each of them. The evaluated attributes are represented on the horizontal axis. The vertical axis represents the information gain value using the Shannon entropy function. At the top of each graph, the trip and the peak time of the trip are indicated. (**a**) entropy of Trip 0–66; (**b**) entropy of Trip 66–0 ; (**c**) entropy of Trip 0–11; (**d**) entropy of Trip 11–0; (**e**) entropy of Trip 99–11; (**f**) entropy of Trip 11–99.

Figure 5 shows the entropy values obtained for the analysed trips and the values obtained for each of the hours of greatest passenger numbers on each of them: 2:00 p.m.–3:00 p.m. on Trip 66–0; 7:00 a.m.–8:00 a.m. on Trip 66–0; 3:00 p.m.–4:00 p.m. on Trip 11–0; 10:00 a.m.–11:00 a.m. on Trip 0–11; 5:00 p.m.–6:00 p.m. on Trip 11–99 and 8:00 a.m.–9:00 a.m. on Trip 99–11. The graphs show that the new generated attributes individually obtain a high information gain value in comparison with the classic time-dependent attributes used as reference.

As a second evaluation criterion, the effect of the new attributes was used to predict demand through neural networks. Specifically, “Resilient Backpropagation with Weight Backtracking” networks were used, due to their rate of convergence. The number of input neurons was determined by the total number of attributes used and the number of neurons in the hidden layer varied between one and five. In all cases, there is an output neuron with the estimated total number of passengers.

In this case, the network training and evaluation procedure was carried out using the cross-validation technique that provides an estimate with low bias [36], using the same records that correspond to the year of study (2015). Specifically, five folders were used. The observed error, *Eo*, in the prediction is the average value of the error obtained, *E*(*i*), for each of the events, *i*, and is calculated as the difference between the normalised values of the real value, *VR*(*i*), and the estimated value *VE*(*i*):

(3) E(i)=|VR(i)−VE(i)|

(4) $$\mathrm{Eo}=\overset{n}{\underset{i=1}{\sum }}\frac{E\left(i\right)}{n}\times 100$$

**Figure 6:**
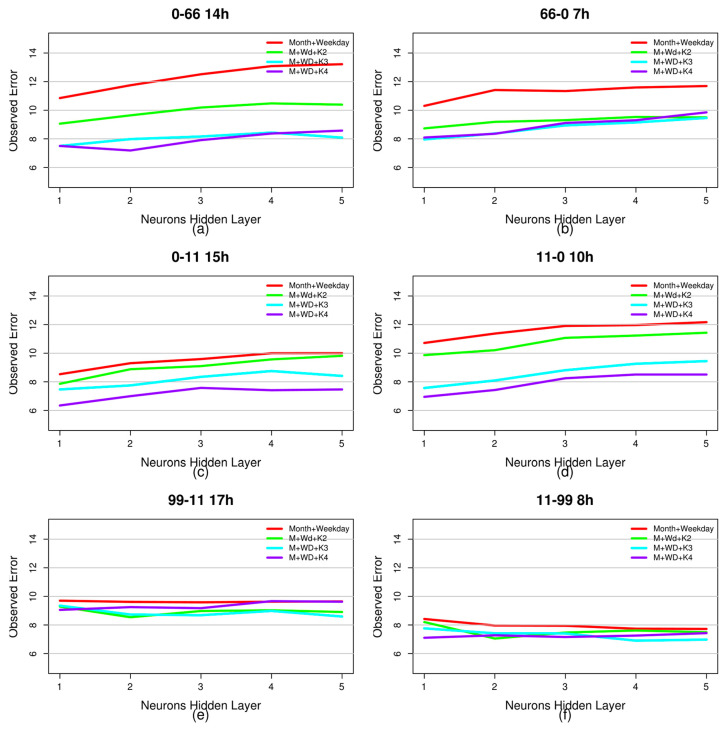
Observed error in the prediction of demand on each of the trips analysed in the peak periods of each one of them. The number of neurons in the hidden layer is represented on the horizontal axis. The vertical axis represents the observed error. At the top of each graph, the trip and peak time for which the prediction was made are indicated. (**a**) error on Trip 0–66; (**b**) error on Trip 66–0 ; (**c**) error on Trip 0–11; (**d**) error on Trip 11–0; (**e**) Error on Trip 99–11; (**f**) Error on Trip 11–99.

Figure 6 shows the errors observed when estimating demand with the month and day of the week attributes and when it was estimated adding each of the new generated attributes to them. As was done with the entropy evaluation, this estimate was made in the time period with the greatest number of travellers on each route analysed. In all cases, it can be seen that the incorporation of the new attribute reduces the error in the estimation; in the case of Trip 0–66, this is close to 40% for the network with five neurons in the hidden layer. Table 4 gives a summary of the aforementioned results, representing the mean observed error on each of the routes for the different neural network configurations used. The baseline was the prediction using only the month and day of the week–attributes that are frequently used in this type of prediction [37]. As can be seen when adding any of the new attributes, the error in the prediction was reduced, in some cases by up to 30%.

When choosing the number of clusters to be used to obtain the new attributes, two mistakes must be avoided: the first is that the number of clusters chosen is greater than the number of clusters that actually exist in the data, and the second that the number of clusters chosen is lower. Although it depends on the context of the problem to be solved, it is worse to commit the latter error since this would lead to a loss of information when the results are interpreted [38]. Considering the above and observing, firstly, that the best consistency value was obtained using two clusters and, secondly, that analysis of the clusters, in terms of the information provided and the error committed in the prediction, shows that better results are obtained with three and four clusters, it was decided that three clusters would be used to interpret the results in the following Discussion section.

## 5. Discussion

**Figure 7:**
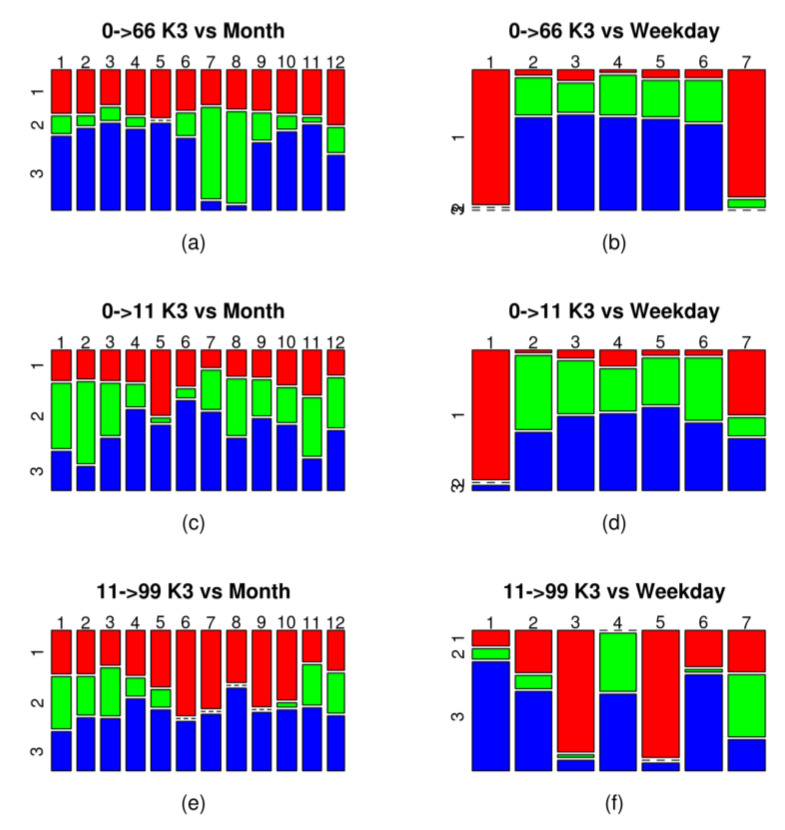
Graphic representation of the contingency table for the groupings of three clusters of three trips of the six that were analysed, according to the month of the year and the day of the week. The horizontal dimension is associated with a period of time (month in the tables on the left and day of the week in the tables on the right). The vertical dimension is associated with the clusters. (**a**) clusters of Trip 0–66 according to the month; (**b**) clusters of Trip 0–66 according to the day of the week; (**c**) clusters of Trip 0–11 according to the month; (**d**) clusters of Trip 0–11 according to the day of the week; (**e**) clusters of Trip 11–99 according to the month; (**f**) clusters of Trip 11–99 according to the day of the week.

Evaluation of the proposed new attributes show that they can provide information in addition to that provided by traditional time-dependent attributes. From the demand patterns, represented by the new attributes, more precise knowledge of demand can be obtained if we analyse each one of the obtained clusters. One way to analyse the results is to use contingency tables to represent the proportion of data from each of the clusters belonging to different periods of time, in order to obtain the types of time periods that significantly affect demand. Figure 7 shows, for the grouping of three clusters, two contingency tables for three of the six trips analysed, one with the months of the year (Figure 7a,c,e) and the second with the days of the week (Figure 7b,d,f). 

It can be seen in Figure 7a that, in the case of Trip 0–66, Cluster 3, which corresponds to the highest demand profile (Figure 4a) is concentrated mainly in 10 months of the year (January–June and September–December). These periods correspond to school periods in which the use of public transport to go to schools pushes up the number of public transport users. For this same route, Cluster 2, which is associated with an average demand profile, is concentrated in the months of July and August, a holiday period in which travel to school decreases. Cluster 1, which corresponds to the lowest demand profile, has a uniform distribution over every month of the year and is concentrated on days of lower working activity (Saturday and Sunday) (Figure 7b).

For Trip 0–11, the interpretation of the new attributes indicates a demand that is different and more complex from the previous patterns. In this case, the cluster with the highest demand profile is Cluster 2, with data records concentrated mainly from January to March, and, in August, November and December (Figure 7c). It is followed by Cluster 3, which is mainly concentrated in the period from April to June. Taking into account the time slots in which demand peaks occur (Figure 4b) and the fact that most travellers use direct payment instead of card payment (Table 3), this behaviour can be interpreted as visitors returning from the city of Las Palmas de Gran Canaria to the tourist centre where they are staying (San Bartolomé), since Cluster 2 corresponds to the months of the high tourist season and Cluster 3 to the low season. This demand can also be explained by the overlapping of two types of high and low season seen in the tourist destination of San Bartolomé de Tirajana. In winter, visitors come mainly from the countries of northern and central Europe, while, in the summer months, domestic tourism predominates. As was the case with the previous trip, a specific demand profile for Saturdays and Sundays reappears, corresponding to Cluster 1 (Figure 7d). 

For Trip 99–11, in the analysis with contingency tables of attribute *K3*, they show that the cluster with the highest demand profile is Cluster 2, which mostly corresponds to the months of January, February, March, November and December (Figure 7e). This behaviour can be interpreted as the flow of travellers arriving at the airport and travelling to their place of accommodation in the tourist high season, which corresponds to visitors from central and northern Europe for whom the use of public transport is a more common habit than it is for domestic tourists. In addition, the contingency table for the days of the week (Figure 7f) shows demand that is different from the two previously mentioned trips. In this case, the days of lowest passenger numbers do not come on the weekend; they are on Tuesday and Thursday. This weekly demand can be interpreted as being related to flight scheduling by the airlines.

**Figure 8:**
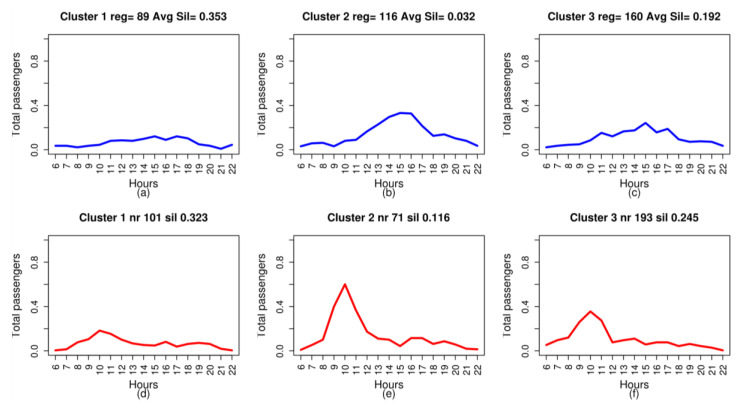
Medoids of three clusters of the two trips between stops 0 and 11. The medoids in blue are those obtained for the route between stop 0 and 11 and the medoids in red are those obtained for the route between stop 11 and 0. The horizontal axis represents the time of day, from 6:00 a.m. to 10:00 p.m. The vertical axis represents the number of passengers, normalised: (**a**) medoid of Cluster 1 on Trip 0–11; (**b**) medoid of Cluster 2 on Trip 0–11; (**c**) medoid of Cluster 3 on Trip 0–11; (**d**) medoid of Cluster 1 on Trip 11–0; (**e**) medoid of Cluster 2 on Trip 11–0; (**f**) medoid of Cluster 3 on Trip 11–0.

Analysis of the medoids of the new attributes also makes it possible to infer information about demand, particularly when jointly studying the medoids of the trips between the pairs of stops under analysis. This analysis sheds light on which stop attracts the most passengers on that route depending on the time and makes it possible to infer what type of passenger makes the trip. Figure 8 shows the three medoids of Trips 0–11 and 11–0, to illustrate this aspect of the discussion. A significant common characteristic of the trips, pertaining to the profiles of greatest demand on these two trips (Figure 8b,c for Trip 0–11 and Figure 8e,f for Trip 11–0), is the fact that, for both trips, the time periods in which the peaks of greatest demand are produced in the two groups coincide. For Trip 0–11, from the main station in Las Palmas de Gran Canaria to the main stop in San Bartolomé, this peak time occurs between 2:00 p.m. and 4:00 p.m. For Trip 11–0, from the main stop in San Bartolomé to the main station in Las Palmas de Gran Canaria, this peak in demand occurs between 9:00 a.m. and 11:00 a.m. This behaviour leads to the conclusion that the typical passenger between these stops travels from stop 11 to 0 between 9:00 a.m. and 11:00 a.m., and returns between 2:00 p.m. and 4:00 p.m. It may also be concluded that this passenger is a visitor who is staying in the tourist town of San Bartolomé and is making a short visit, of between three and five hours, to the city of Las Palmas de Gran Canaria; the duration of the visit matches the visitor’s time habits. This interpretation, based on the cluster medoids, is coherent and reinforces the interpretation of the contingency tables for this route: associating the periods in which the two patterns of greatest demand were produced with the island’s two tourist seasons.

With regard to the prediction made using the new attributes, this was done using cross-validation since the main objective was to evaluate them and the method used to generate them. However, the results are promising since the incorporation of the new attributes reduces error when compared to traditional attributes, with the exception of two configurations of the network on Trip 99–11, where this error is equal.

## 6. Conclusions

This paper proposes a new type of time-dependent attribute to classify demand on public road transport systems. Moreover, the methodology that was followed has been described and implemented to facilitate the systematic generation of these attributes. The methodology is based on data mining.

From the results that were obtained from implementation in a real use case of demand analysis in a public passenger transport company, it was concluded that the proposed new attributes provide more information than the time-dependent attributes that have traditionally been used to design transport networks and to schedule public road transport systems. In addition, these new attributes enable us to classify demand over different time scales, taking into account different factors, and to obtain information on aspects such as the stops that attract the most passengers, type of traveller and reason for the trip. 

The results have demonstrated the suitability of the methodology for the systematic acquisition of these new attributes. It is based on data mining and, more specifically, on clustering techniques, entropy analysis and neural networks. Finally, this methodology can be implemented in most intercity public transport companies, since it uses data that are usually found in companies’ information systems, so it does not require external data sources. 

## Figures and Tables

**Table 1:** Structure of the dataset for classifying demand.

*P_o_*	*P_d_*	*W*	*D*	*A_W,D,H0_*	*A_W,D,H1_*	*A_W,D,H2_*	......................................	*A_W,D,Hf_*

**Table 2:** Structure of the data records for the training and test dataset.

*P_o_*	*P_d_*	*W*	*D*	*F_0_*	*F_d_*	*H*	*C*	*N*

**Table 3:** Number of passengers on each trip according to the means of payment used.

Trip O–D	Direct Payment Passengers	Card Payment Passengers
Trip 0–66	53,067	34,127
Trip 66–0	41,701	23,848
Trip 0–11	135,715	17,173
Trip 11–0	125,181	15,074
Trip 99–11	73,026	2086
Trip 11–99	76,214	2251

**Table 4:** Mean observed error for the different configurations of the neural network (number of units in the hidden layer).

	Trip (Peak Period)					
Attributes	0–66 (14 h)	66–0 (7 h)	0–11 (15 h)	11–0 (10 h)	99–11 (17 h)	11–99 (8 h)
Month and Day of the week (baseline)	12.28	11.27	9.49	11.63	9.64	7.96
Month, Day of the week and *K*2	9.95	9.25	9.04	10.76	8.95	7.57
Month, Day of the week and *K*3	8.03	8.78	8.15	8.64	8.87	7.29
Month, Day of the week and *K*4	7.91	8.94	7.16	7.93	9.35	7.25
